# Thickness determination of the tidemark of human articular cartilage using high-resolution micro-XRF imaging of zinc and lead^☆^

**DOI:** 10.1016/j.ocarto.2021.100182

**Published:** 2021-05-26

**Authors:** M. Rauwolf, A. Turyanskaya, P. Wobrauschek, K. Sawhney, A. Roschger, P. Roschger, C. Streli, J.G. Hofstaetter

**Affiliations:** a Atominstitut, TU Wien, Vienna, Austria; b Diamond Light Source Ltd, Harwell Science & Innovation Campus, Didcot, Great Britain, UK; c Paris-Lodron-University of Salzburg, Department of Chemistry and Physics of Materials, Salzburg, Austria; d Ludwig Boltzmann Institute of Osteology at the Hanusch Hospital of OEGK and AUVA Trauma Centre Meidling, Vienna, Austria; e Michael Ogon Laboratory for Orthopaedic Research, Orthopaedic Hospital Vienna-Speising, Vienna, Austria

**Keywords:** Tidemark, Zinc, Lead, SR micro-XRF, Elemental imaging

## Abstract

**Objective:**

The objective of the study was to specify the thickness of Zn and Pb accumulation within the tidemark (TM), a narrow structure between the non-calcified and the calcified articular cartilage. It is considered an active or resting calcification front. This banded structure of the cartilage-bone interface is known to undergo changes in osteoarthritis. Therefore, gaining knowledge about this structure is of interest.

**Methods:**

Femoral head samples were collected from patients suffering from various bone diseases, 6 samples have been investigated. Thin bone slices (3 ​μm thick) were measured with high resolution synchrotron micro-X-ray fluorescence (SR micro-XRF) analysis using a beam with dimensions of 500 ​× ​800 ​nm^2^. The tidemark region was found in all analyzed samples. The Savitzky-Golay filter was used to smooth the measured imaging data and Kaplan-Meier estimation to gain reliable tidemarks medians for Pb and Zn. To our knowledge this was the first time that these methods have been applied to gain information on histological structures obtained by elemental imaging.

**Results:**

The thickness of the Zn and Pb layer ranged from about 3 to 11 ​μm for Zn and 4–14.5 ​μm for Pb. Our Zn ratios (TM/matrix) were found to be 1.5-3-fold ratio between Zn tidemark values and in mineralized matrix and are similar in all samples.

**Conclusions:**

The determined thickness of the layer is much smaller than found in previous measurements with the beam having 20 ​× ​14 ​μm^2^ size. The Zn ratios agree with our previous findings.

## 1 Introduction

The tidemark is a narrow zone at the border of non-calcified and calcified articular cartilage and is considered an active or resting calcification front [2]. Double and multiple tidemarks can be observed indicating that new phases of mineralization have started with a new mineralization front, surrounding the old tidemark with newly mineralized cartilage matrix [3]. This banded structure of the cartilage-bone interface is known to undergo changes in osteoarthritis [[4], [5], [6]].

Using synchrotron micro-X-ray fluorescence (SR micro-XRF) measurements with lower resolution (beam size about 20 ​μm ​× ​14 ​μm) our group has previously reported that Zn and Pb accumulate in the tidemark region [3,7]. However, as the layer of Zn and Pb accumulation was suspected to be thinner than the diameters of the used X-ray beams, the true local concentrations would be underestimated.

For this reason, sub-micrometer resolution techniques were applied to gain a better insight into the accumulation of Zn and Pb in the tidemark, its structure and different element levels compared to the mineralized cartilage. The elemental distribution in thin bone samples from 5 different patients was measured.

## 2 Materials and methods

The study was approved by the ethics committee of the Medical University of Vienna, Austria and was done in accordance with the Helsinki Declaration.

The femoral heads were collected from three patients with osteoporotic femoral neck fractures as causative factor for surgery (hemiarthroplasty), P1–P3: all female, 76, 70 and 73 years old, respectively. Further, femoral heads were collected from patients P4 (female, 33 years old, idiopathic osteonecrosis) and P5 (male, 36 years old, osteoarthritis hip).

### 2.1 Sample preparation

From all femoral heads the samples containing articular cartilage as well as sub-chondral bone were prepared. The location was chosen in order to obtain a section plane perpendicular to the plane of the hyaline cartilage in the center of the femoral head so that the sample contains non-mineralized and mineralized cartilage and subchondral bone. The samples were dehydrated and fixed in a gradient of ethanol concentration (50–100%) and afterwards fixed in Polymethyl methacrylate (PMMA), for details see Ref. [8]. Then, six 3 ​μm thick cuts (two cuts from P1) were prepared with a microtome. The sections are further referred to as TM1-6, the numbering corresponds to the patients, with the only exception of P1, with two cuts TM1 and TM6 (see also Table 1). Each of the slices was put between two 8 ​μm thick Kapton foils and fastened to Plexiglas frames. During the measurements, the samples were fixed to a 3D-printed samples carrier so that the frame opening would overlap with the hole in the sample carrier.

**Table 1:** The width of the Zn and Pb layer in all measured sections. Widths (medians of FWHM) in μm. Zn and Pb medians for both tidemark (TM) and mineralized matrix (matrix) are in counts in 20s. The letters i and o indicate inner and outer tidemark (TM). Values in brackets are for higher percentiles (%). These are given in the case where a matrix median of the Kaplan-Meier estimate cannot be given. If available for both TM and mineralized matrix, ratios are calculated using the median. Otherwise the values for the higher percentile was used.

Patient	Section	# of TMs	Width Zn median Zn Pb TM matrix				Ratio Zn	Pb median TM matrix		Ratio Pb
P1	TM1	2	3.0(i) 3.0(o)	4.0(i) 4.0(o)	2274(i) 2182(o)	1246	1.8(i) 1.8(o)	526(i) 104(o)	60	8.8(i) 1.7(o)
	TM6	1	11.0	14.5	2672	937	2.9	753	41	18.4
P2	TM2	1	10.0	9.0	3167	1605	2.0	282	76	3.7
P3	TM3	1	5.0	6.0	3098	1018	3.0	290 (377 for 75%)	- (29 for 75%)	13.0
P4	TM4	2	6.0(i) 9.0(o)	6.5(i) -(o)	3841(i) 2784(o)	1819	2.1(i) 1.5(o)	92 (182 for 95%) (i) -(o)	- (57 for 95%)	3.2(i) -(o)
P5	TM5	1	8.0	9.0	6913	4445	1.6	95 (119 for 75%)	- (42 for 75%)	2.8

### 2.2 Submicro-XRF measurements

**Fig. 1:**
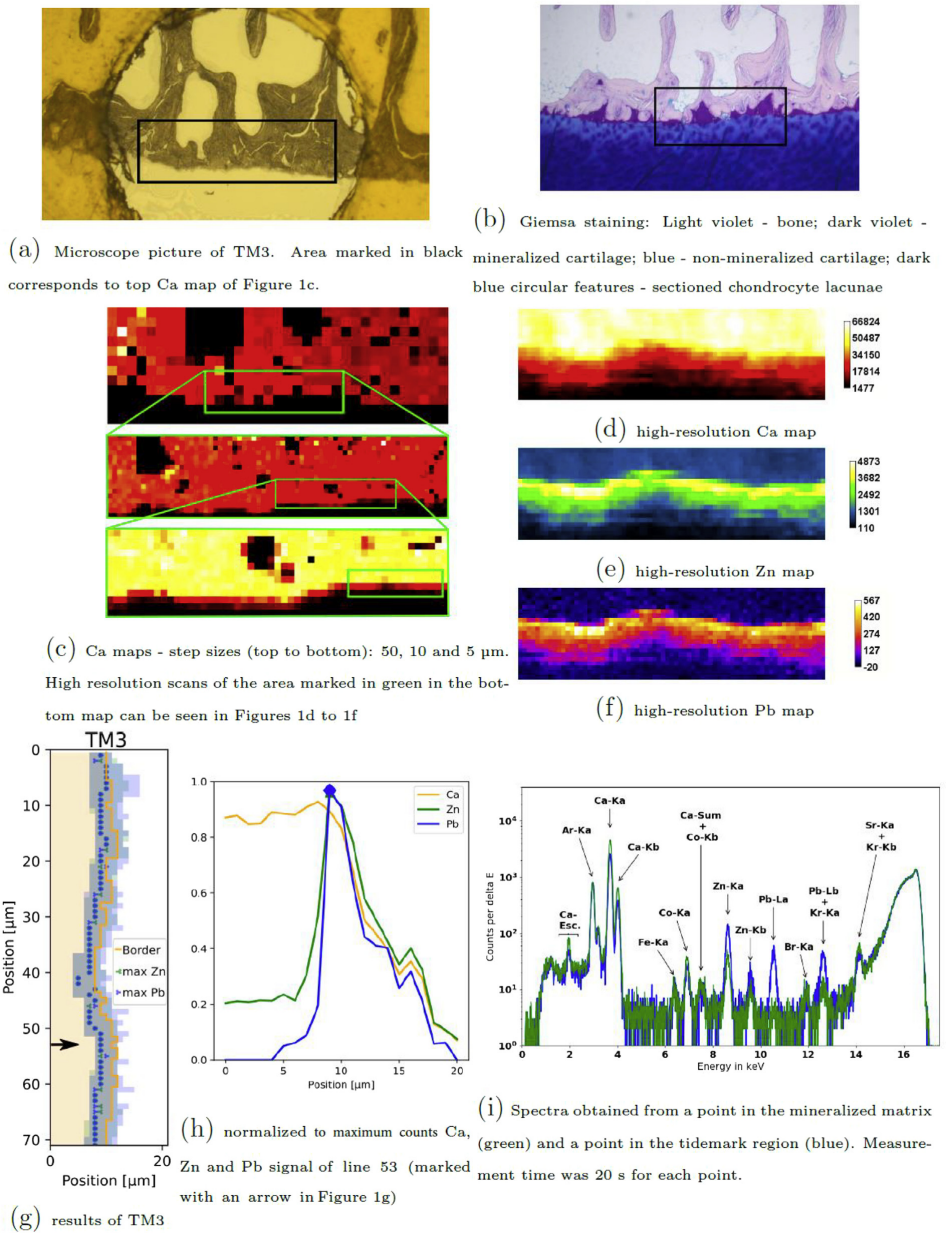
Section TM3. (a) Microscope picture and(b) Giemsa staining of a consecutive cut of the same sample. (c) Ca overview scans used to define the scan position on TM3 and tidemark region scan for(d-f) Ca, Zn and Pb – results in counts/20 ​s. (g) Analysis results: Mineralized matrix (light yellow), border between mineralized and non-mineralized matrix (yellow line), maximum peak position of Zn (green dots) and Pb (blue dots), FWHM for Zn and Pb (semitransparent green and blue, respectively), (h) and of a single scan line of TM3. (i) Typical submicro-XRF spectra.

The samples were measured at the B16 beamline at the Diamond Light Source (DLS), Oxfordshire, UK, with the setup and settings described in Ref. [9]. The size of the exciting 17 ​keV beam was about 500 ​× ​800 ​nm^2^. To find the tidemark area on the samples and to correlate the measured areas with the microscope images of the samples, rather big sample regions were roughly scanned with respect to the Ca signal (1 ​μm step size, 1 ​s measurement time per point) as Ca represents bone. Step size and sample area size were further reduced until a reasonable sized region for the high-resolution scan was found (see Fig. 1a and c). For each sample this high-resolution scan (with 1 ​μm step size) was recorded around the tidemark region. These scans were performed with a measurement time of 20 ​s per point (see Fig. 1d–f).

### 2.3 Data evaluation

The spectra acquired in each point were processed using AXIL software [10]. Net counts per Ca and Zn were converted to text maps (elemental maps). Additionally, also the standard deviation of the fit of each element was converted into a text map.

Custom made software was written in python (v.3.4) for further data processing (see Sections 2.4, 2.5) using the modules: numpy, scipy, matplotlib and lifelines [11].

### 2.4 Identifying Zn and Pb layer in the tidemark region

The following evaluation steps were performed to find the single and double tidemarks within the elemental maps:

1. Data from the Ca, Zn and Pb maps as well as their standard deviations (from the fit mentioned in Section 2.3) are loaded into the program.

2. Ca levels are similar in mineralized cartilage and bone, therefore we do not distinguish between those two and will refer to them as mineralized matrix. The median of the first 5 points of all scan lines within a Ca map were used to represent the Ca level in the mineralized matrix (*Ca*_*mineralized*_). The median of the last 5 data points of each line were used to determine the Ca level non-mineralized cartilage (*Ca*_*non*−*min.*_). As border between the mineralized and the non-mineralized cartilage the following value was defined:
  (1) $$Border=Ca_{non-min.}+0.5\cdot (Ca_{mineralized}-Ca_{non-min.})$$
3. Using the Savitzky-Golay filter (a data smoothing method based on a least-squares polynomial approximation) [12,13] of the scipy module, the Zn and the Pb map data are smoothed line-by-line and the first derivative is estimated to identify the local extrema (minima and maxima). Extrema were only considered to be of interest if the value of positive or negative elevation between them was bigger than 3 times their standard deviation. This restriction was needed otherwise statistical fluctuations are registered as extrema. A peak was defined as two minima with a maximum in-between them. An exception was made for negative elevations in the beginning of a line. For such an elevation to be considered as part of a peak it needed to be greater than or equal to 5 times the standard deviation. Positions of the minima were saved as limits for the search of the actual peaks. The higher value of two consecutive minima was considered the baseline of the peak.

4. Using the position of two consecutive minima in the smoothed data, the maximum (height and position) in-between those minima is determined in the unfiltered data. Using the baseline and the height, the full width at half maximum (FWHM) of the peak is calculated.
5. A peak was accepted as part of a specific tidemark if the position of its FWHM somewhat overlapped with the FWHM of the previous or following line. Peaks which did not satisfy this condition were considered to be part of another possible tidemark.
6. After identifying all potential tidemarks, all candidates shorter than 5 lines were removed from further consideration. This step was done to exclude possible hotspots (location with a high signal).

As an example of the obtained information about the tidemark from this program see the results of one thin cut in Fig. 1g and h.

### 2.5 Kaplan-Meier estimation

As the measurements were optimized to obtain a good Zn signal, a lot of scanned pixels showed no presence of Pb either because the regions contained no Pb or because their concentrations were below the detection limit. As the information about Pb is unavailable on the side of the lower concentrations (which are displayed on the left side in our plots), the data is therefore left censored. The calculation of mean of the fitted values is incorrect for censored data. Ignoring the pixels without Pb in the mean calculation would overestimate the Pb content. The calculation of the mean value might underestimate the Pb content, if one assumes that all pixels that show no presence of Pb (because they are below the detection limit) do not contain any Pb. Therefore, instead of the mean, the median is used as the quantiles are stable location measures (as they are more resistant to outliers), they are suitable parameters to compare samples as long as they are not below the censor level themselves.

M. Pajek et al. have previously shown that the Kaplan-Meier (KM) [14] approach can be used to estimate the cumulative distribution function from left-censored XRF data fairly accurately [15]. The KM algorithm creates a curve very similar to an empirical cumulative distribution function. If there are no censored values, the KM curve becomes the empirical cumulative distribution function.

**Fig. 2:**
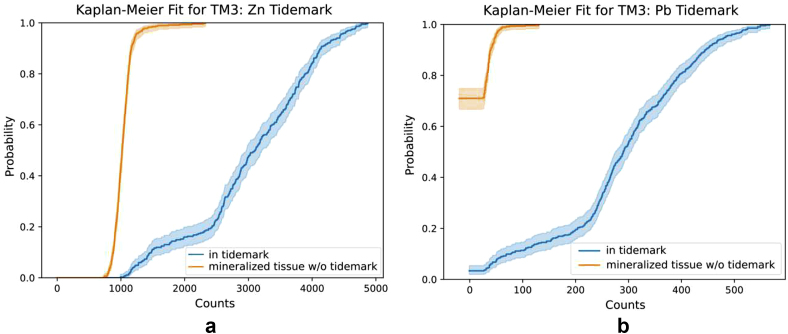
Kaplan-Meier estimate for Zn (a) and Pb (b) within the mineralized tissue (orange) and the tidemark (blue).

Fig. 2a shows the KM-estimated cumulative distribution function for Zn in the tidemark and the remaining calcified tissue of the measured area of TM3 slice, as presented in Fig. 1. The medians for those two groups can easily be compared by looking at the 0.5 probability level. The Zn median is about 3100 counts for the tidemark and 1020 counts for the mineralized tissue, respectively. As can be seen in Fig. 2b, no Pb was detected in about 70% of the mineralized tissue, and the Pb median value within the tidemark is ca. 300 counts. Median counts for all the tidemarks are provided in Table 1.

## 3 Results and conclusions

Tidemark regions were identified and measured in areas of all six measured bone tissue sections (the resultant overlays for all six sections are included in the supplementary material).

**Fig. 3:**
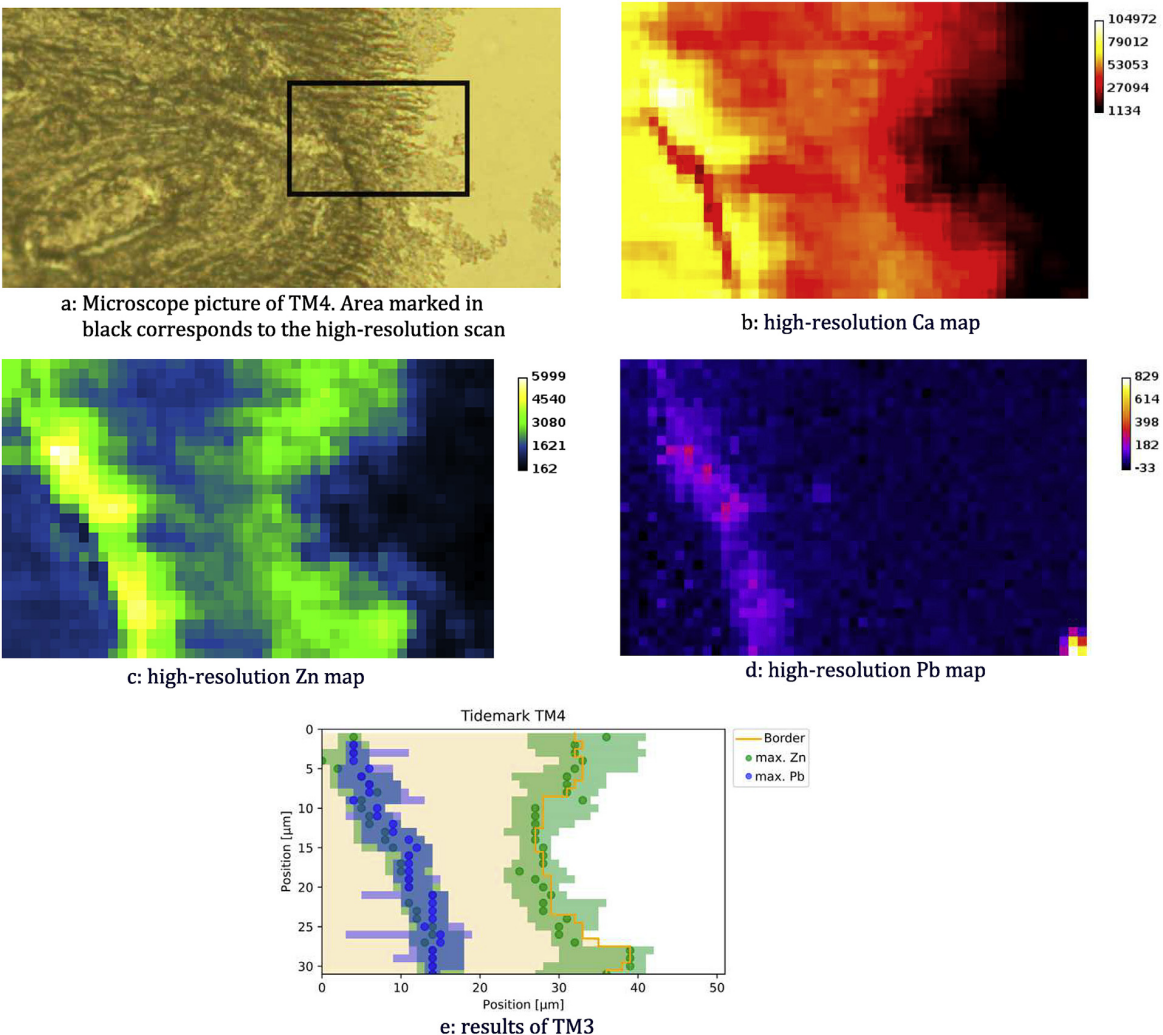
Section TM4, example of double Zn tidemark. (a) Microscope picture and(b-d) tidemark region scan for Ca, Zn and Pb – results in counts/20 ​s. (e) Analysis results: Mineralized matrix (light yellow), border between mineralized and non-mineralized matrix (yellow line), maximum peak position of Zn (green dots) and Pb (blue dots), FWHM for Zn and Pb (semitransparent green and blue, respectively).

In two of the measured sections (TM1 and TM4, corresponds to P1 and P4, respectively) the elemental map exhibits two Zn lines indicating the presence of a double tidemark [3], an example can be seen in Fig. 3. In sample TM1, Pb was also found within these two tidemarks while TM4 only exhibited a Pb signal in the inner one. Mineralized matrix medians for Zn ranged from about 900 to 1800 counts in 20 ​s for most samples (patients P1-4). For the same patients, the tidemark Zn medians ranged from about 2200 to 3800 counts in 20 ​s. The patient with significantly higher median values (about 6900 counts in 20 ​s for the tidemark and 4400 for the matrix) was P5 (the only male patient). For only two patients (P1 and P2) a median Pb count value could be detected within the mineralized matrix. For all other patients, Pb did not have a detectable KM median (due to the left-censored data). Median Pb tidemark values ranged from 92 to 526 counts in 20 ​s.

The median width (FWHM) of the tidemarks ranged from 3 - 11 and 4–14.5 ​μm for Zn and Pb, respectively. The widths for all analyzed areas are listed in columns “Median width (FWHM in μm)” of Table 1. Interestingly, the highest differences were found between the two cuts of the same patient (P1). Median counts for the tidemarks can also be found in Table 1. Three samples had such low counts above the detection limit in the mineralized tissue that the medians could not be compared. For those samples higher percentiles were given.

Consistent with previous findings [3], similar Zn levels in the inner and outer tidemark were found while Pb levels were significantly higher in the inner tidemark. The Zn ratios (TM/matrix) were similar in all samples and were found to be within 1.5-3-fold range, which fits well to the previous findings reported as 3-5-fold ratio between Zn tidemark values and in mineralized matrix [3,16]. Pb showed a higher variability over all samples which might be explained by the individual history of Pb exposure of the patients. This is also consistent with our previous findings. Depending on how high the Pb and Zn levels in the tidemark are, it might be possible that during joint erosion (for example in osteoarthritis patients) the systemic levels of Zn and Pb might increase to values of medical relevance.

Tidemark thickness ranged from about 3 to 11 ​μm for Zn and 4 to 14.5 ​μm for Pb. As a result of the increased resolution these values are now much more reliable compared to previous estimations of about 40 ​μm [16].

## Author contributions

MR and AT performed the measurements at Diamond Light Source. MR carried out the statistical data evaluation. PR and AR prepared the samples. KS provided support at the B16 beamline at DLS. All authors have made a substantial contribution to the concept and design of this study, interpretation of the data, drafting and revising the content of this article, and approved the final version being submitted.

## Role of the funding source

This study is funded by a Project P27715 from 10.13039/501100002428FWF. The funding source was not involved in the study design, in the collection, analysis, and interpretation of the data, in the writing of this manuscript, or the decision to submit this manuscript for publication.

## Declaration of competing interest

All authors declare no conflicts of interest.
