# Supplementary figures and images for: Thickness determination of the tidemark of human articular cartilage using high-resolution micro-XRF imaging of zinc and lead
Source: Osteoarthr Cartil Open. 2021 May 26;3(3):100182. doi: 10.1016/j.ocarto.2021.100182 (PMC9718329; doi:10.1016/j.ocarto.2021.100182)

TM1

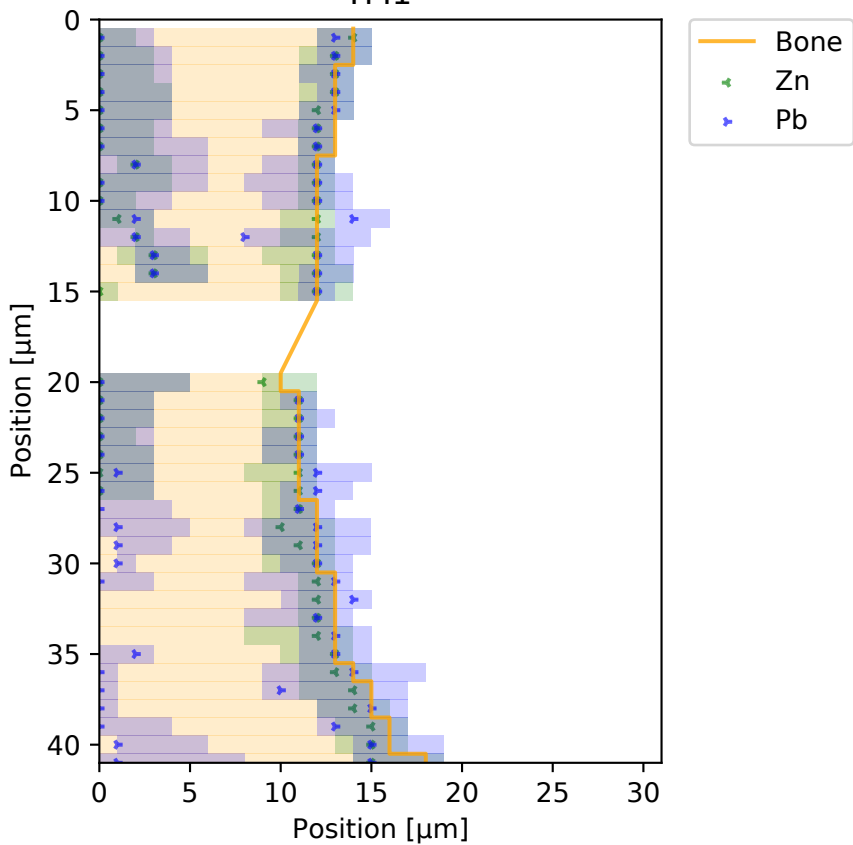

Supplement: Multimedia component 1 [file mmc1.pdf]

TM2

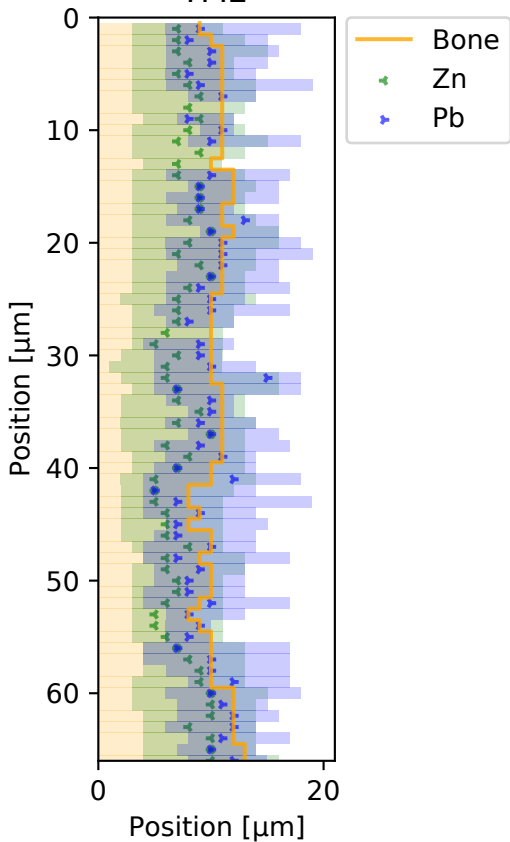

Supplement: Multimedia component 2 [file mmc2.pdf]

# TM3

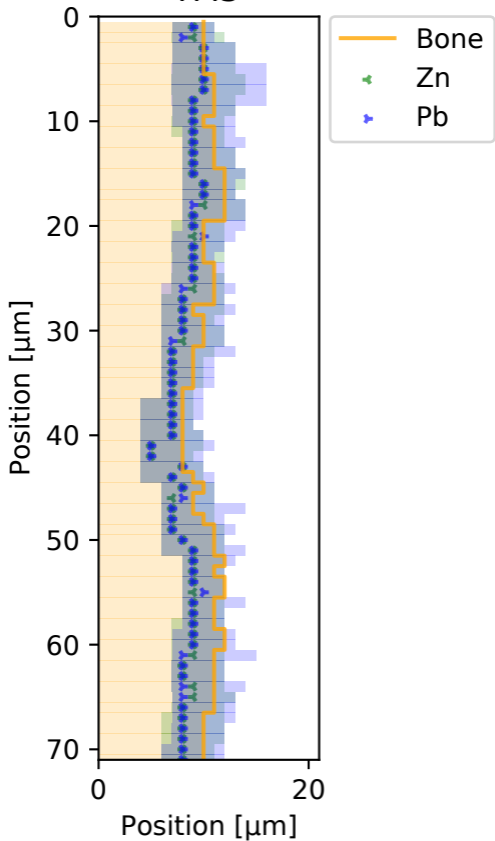

Supplement: Multimedia component 3 [file mmc3.pdf]

TM4

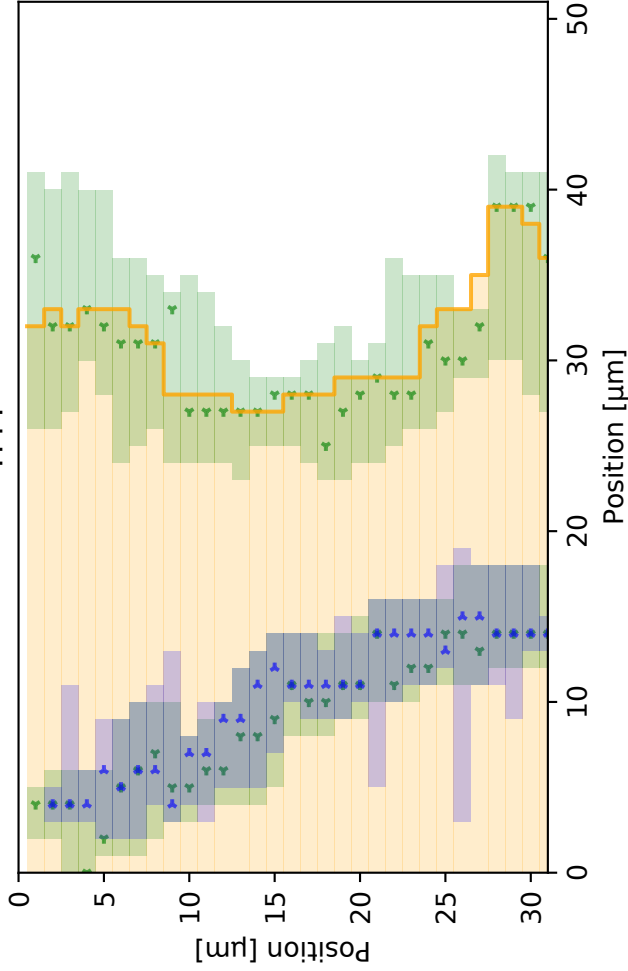

Supplement: Multimedia component 4 [file mmc4.pdf]

# TM5

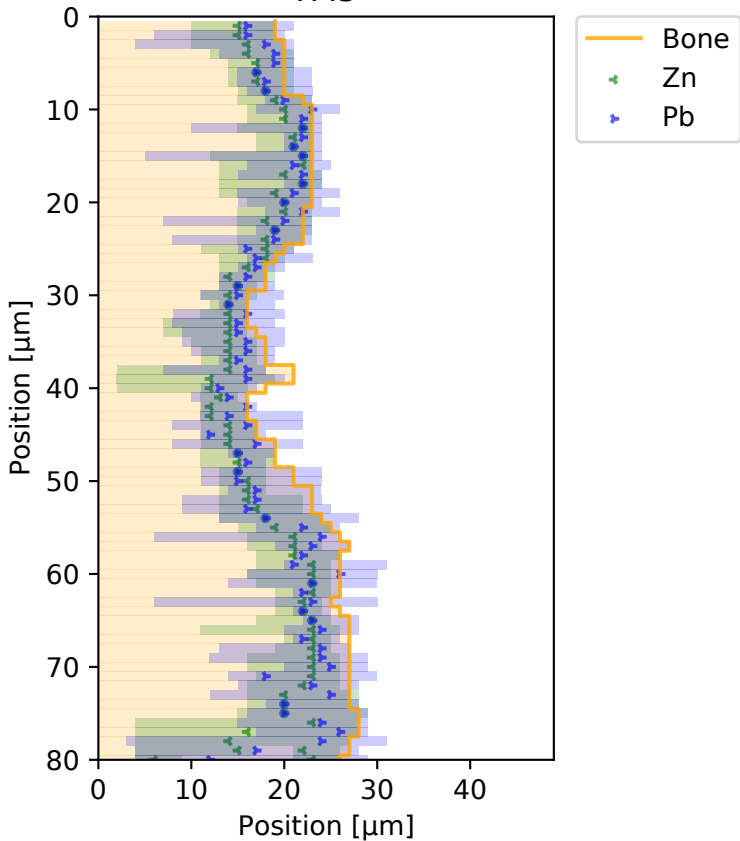

Supplement: Multimedia component 5 [file mmc5.pdf]

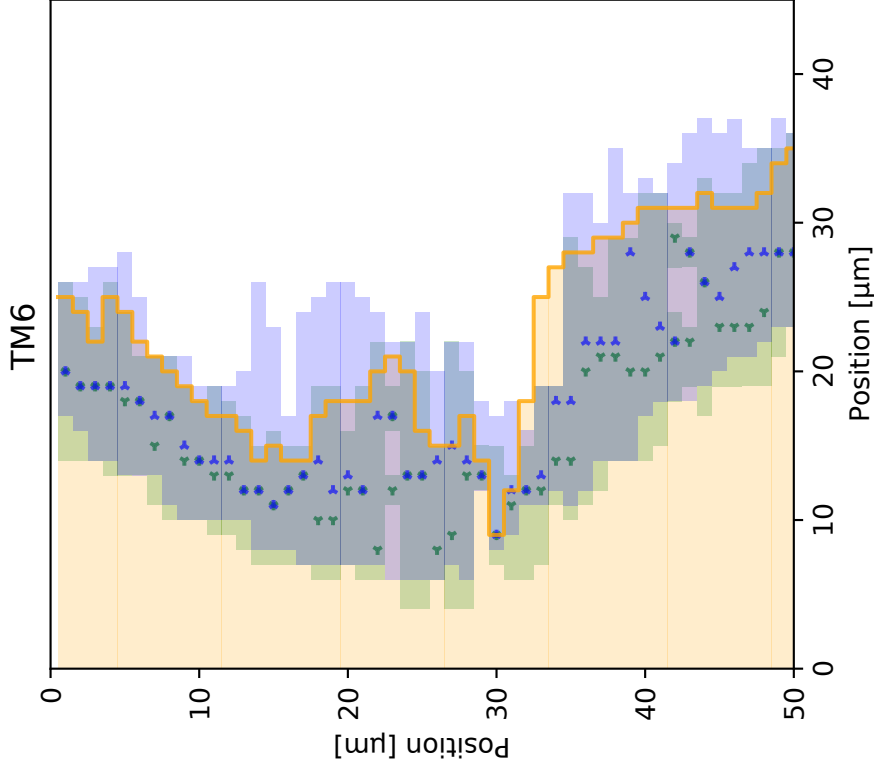

Supplement: Multimedia component 6 [file mmc6.pdf]
